# Integrating High-Throughput Approaches and *in vitro* Human Trophoblast Models to Decipher Mechanisms Underlying Early Human Placenta Development

**DOI:** 10.3389/fcell.2021.673065

**Published:** 2021-06-02

**Authors:** Bum-Kyu Lee, Jonghwan Kim

**Affiliations:** ^1^Department of Biomedical Sciences, Cancer Research Center, University at Albany-State University of New York, Rensselaer, NY, United States; ^2^Department of Molecular Biosciences, Center for Systems and Synthetic Biology, The University of Texas at Austin, Austin, TX, United States

**Keywords:** placenta, trophoblast, trophoblast stem cells, human placenta models, transcriptomes, epigenomes, omics approaches

## Abstract

The placenta is a temporary but pivotal organ for human pregnancy. It consists of multiple specialized trophoblast cell types originating from the trophectoderm of the blastocyst stage of the embryo. While impaired trophoblast differentiation results in pregnancy disorders affecting both mother and fetus, the molecular mechanisms underlying early human placenta development have been poorly understood, partially due to the limited access to developing human placentas and the lack of suitable human *in vitro* trophoblast models. Recent success in establishing human trophoblast stem cells and other human *in vitro* trophoblast models with their differentiation protocols into more specialized cell types, such as syncytiotrophoblast and extravillous trophoblast, has provided a tremendous opportunity to understand early human placenta development. Unfortunately, while high-throughput research methods and omics tools have addressed numerous molecular-level questions in various research fields, these tools have not been widely applied to the above-mentioned human trophoblast models. This review aims to provide an overview of various omics approaches that can be utilized in the study of human *in vitro* placenta models by exemplifying some important lessons obtained from omics studies of mouse model systems and introducing recently available human *in vitro* trophoblast model systems. We also highlight some key unknown questions that might be addressed by such techniques. Integrating high-throughput omics approaches and human *in vitro* model systems will facilitate our understanding of molecular-level regulatory mechanisms underlying early human placenta development as well as placenta-associated complications.

## Introduction

As a transient but multifunctional organ essential for the proper development of the fetus in placental mammals, the placenta plays a central role in multiple processes during pregnancy, such as gas and nutrient exchange, hormone production, and immunological protection (Rossant and Cross, 2001). Despite these important roles, the placenta has not received sufficient attention, remaining one of the least studied organs in the body (Cao and Fleming, 2016). It is noteworthy that a recent large-scale mouse knockout (KO) study has revealed that 68% of lethal mouse lines show morphological abnormality of the placenta (Perez-Garcia et al., 2018). The prevalence of placental deformities in KO of embryonic lethal genes emphasizes the significance of the placenta for the proper development of embryos, although this has not yet been systematically confirmed in humans.

The placenta originates from the trophectoderm (TE) of the blastocyst stage of the developing embryo, and it consists of multiple trophoblast cell types, including cytotrophoblasts (CT) and more specialized syncytiotrophoblasts (ST) and extravillous trophoblasts (EVT) in humans (Perez-Garcia et al., 2018). Abnormal trophoblast lineage development results in placental dysfunctions, which can cause morbidity and mortality in both mother and fetus. Defective placentas not only contribute to maternal insulin resistance, preeclampsia (PE), and gestational hypertension, but also result in premature growth of the fetus. These adverse effects often persist long after birth and predispose offspring to various chronic adult disorders, such as diabetes and cardiovascular and mental diseases (Hales and Barker, 2001; Barker and Thornburg, 2013; Courtney et al., 2018). Although the etiologies of pregnancy disorders are often multifactorial, prior research has suggested a direct link between the defect in trophoblast differentiation and pregnancy-related complications, such as PE and intrauterine growth restriction (IUGR) (Chen et al., 2002; Ergaz et al., 2005; Uzan et al., 2011).

Although the placenta is essential, limited access to the human placenta, particularly during the early stages of pregnancy, has hindered the molecular-level understanding of both normal and abnormal placenta development. For many years, trophoblast carcinoma cells, primary CT, and mouse or rat trophoblast stem cells (TSCs) have been used as *in vitro* models for trophoblast differentiation despite some drawbacks (Nagamatsu et al., 2004; Bilban et al., 2010; Latos and Hemberger, 2016; Dietrich et al., 2020), Mouse TSCs (mTSCs) have been extensively studied in combination with various research tools, including high-throughput approaches, revealing numerous key regulators, including transcription factors (TFs) and their regulatory mechanisms, and enhancing our understanding of general trophoblast development (Prudhomme and Morey, 2016; Lee et al., 2019; Ullah et al., 2020). Nevertheless, as human and mouse pregnancy do not share all physiological features, and recently established human TSCs (hTSCs) do not robustly express several previously known mTSC-specific key regulators such as *Cdx2, Eomes, Esrrb*, and *Sox2* (Okae et al., 2018), there is a pressing need to utilize human model systems to better understand human-specific trophoblast lineage differentiation and placentation.

**FIGURE 1:**
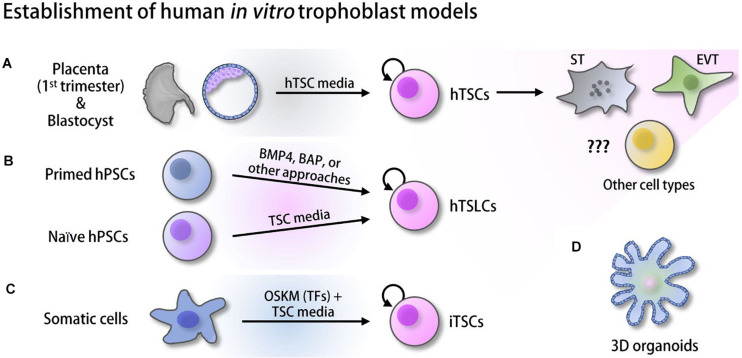
Derivation of human *in vitro* placenta models. **(A)** Human trophoblast stem cells (hTSCs) derived from the trophectoderm (TE) of the blastocysts and first-trimester placenta (Okae et al., 2018). **(B)** Trophoblast stem-like cells (TSLCs) derived from various defined media (Xu et al., 2002; Amita et al., 2013; Li et al., 2013, 2019; Horii et al., 2016; Mischler et al., 2021). **(C)** Induced trophoblast stem cells (iTSCs) reprogrammed from somatic cells using ectopic expression of OCT4, SOX2, KLF4, and MYC (OSKM) followed by culture in hTSC media (Castel et al., 2020; Liu et al., 2020). **(D)** 3D organoids established from hTSCs and cytotrophoblast (CT) (Haider et al., 2018; Turco et al., 2018; Saha et al., 2020).

Only recently, *bona fide* hTSCs, TS-like cells (TSLCs) that can mimic hTSCs in some aspects, and induced TSCs (iTSCs) have been established from various sources of human cells and started to gain attention for their utility (Figure 1). Some of these cells self-renew and retain a capacity to differentiate into multiple specialized cell types, such as ST and EVT (Okae et al., 2018; Castel et al., 2020; Dong et al., 2020; Liu et al., 2020). However, these *in vitro* human trophoblast model systems are relatively new and therefore have not been extensively studied yet. As various high-throughput omics approaches in the field of pluripotent stem cells (PSCs) have aided in understanding early embryo development by identifying critical *cis*- and *trans*-regulatory factors and their regulatory mechanisms (Loh et al., 2011), such omics approaches in combination with *in vitro* human placenta models will provide us with clues for the understanding of human placentation. In this review, we first provide a broad overview of multiple omics approaches used in the studies of mTSCs or other fields and key outcomes, and briefly describe differences between human and mouse placentation. Since most studies have been performed in mTSCs, the vast majority of data we reviewed here are from mouse studies, with occasional studies from human trophoblast and the placenta. Then, we introduce recently reported *in vitro* human trophoblast model systems and their applications, collectively emphasizing the pressing needs of similar omics approaches to be applied to human *in vitro* models to enable identification of previously unknown human trophoblast-specific key factors and their regulatory mechanisms underlying both normal and abnormal human placenta development.

## Various Omics Studies on Placenta/Trophoblast-Specific Modulators and Their Regulatory Mechanisms

Transcriptional and epigenetic regulations govern global gene expression programs, thereby modulating cellular functions and identity *via* the interactions between *cis*-regulatory elements, such as promoters, enhancers, and insulators, and numerous *trans*-acting factors, including cell-type-specific TFs and cofactors. Recent next-generation sequencing (NGS)-based genomics studies enable us to exhaustively determine cell-type-specific TFs, enhancer-TF interactions, and TF-TF interactions responsible for precise cellular functions in various contexts. Additionally, genome-wide profiling of histone modifications, DNA methylation landscapes, chromatin accessibility, and three-dimensional architectures has advanced our understanding of the mechanisms underlying cellular identity and animal development. In the field of trophoblast biology, due to the late establishment of human *in vitro* models, most omics studies have been performed in mTSCs in addition to *in vivo* functional studies in mouse KO models. In this section, we review some important omics approaches, such as chromatin immunoprecipitation coupled with high-throughput sequencing (ChIP-seq), transposase-accessible chromatin followed by sequencing (ATAC-seq), high-throughputpromotercapture (Hi-C), and other approaches mainly taken in mTSCs or other contexts. Integrative studies with *in vitro* human trophoblast models and various omics approaches will facilitate our understanding of the mechanisms underlying early human placenta development.

### Mapping Cis-Regulatory Elements in mTSCs and Mouse Placenta

As ChIP-seq allows for the identification of pivotal *cis*-regulatory elements controlling tissue-specific gene expression, multiple ChIP-seq studies of an enhancer-binding protein (p300) or enhancer-associated histone marks (H3K27ac and H3K4me1) have identified comprehensive sets of enhancers in mTSCs and mouse placentas (Shen et al., 2012; Chuong et al., 2013; Tuteja et al., 2016; Lee et al., 2019). Integrative analyses of p300, H3K4me1, and H3K27ac ChIP-seq data sets have identified approximately 70K putative enhancers in mouse placenta, of which 4,431 enhancers were placenta-specific among 19 tissues tested (Shen et al., 2012). Placenta-specific enhancers often have stage-specific roles during placentation and may be implicated in certain placenta complications. For example, profiling of global enhancers and transcriptomes at the pinnacle and shortly after the trophoblast invasion revealed that many active enhancers contain three enriched motifs of trophoblast-specific TFs (AP1, ETS2, and TFAP2C), suggesting that these enhancer-TF networks may play essential roles in controlling the depth of trophoblast invasion during placenta development (Tuteja et al., 2016). Therefore, mapping enhancer-TFs networks will facilitate understanding of proper trophoblast invasion, which is essential for advancing treatments for pregnancy complications caused by aberrant trophoblast invasion, including placenta accreta (due to too much invasion) and PE as well as IUGR (due to too shallow or incomplete invasion) (Caniggia et al., 2000; Tantbirojn et al., 2008; Barrientos et al., 2017).

Interestingly, global mapping and comparison of enhancers between mouse and rat TSCs unveiled that endogenous retroviruses (ERVs) are strongly enriched within the species-specific enhancers among transposable elements. In particular, the ERV known as RLTR13D5 contributes to the mouse-specific enhancer landscape, suggesting differential insertions of transposable elements within the genome of various species may lead to placental diversity by altering the binding sites of TSC-specific TFs, such as ELF5, TFAP2C, and TEAD4 (Chuong et al., 2013). However, much remains unknown concerning the contribution of transposable element-associated enhancers to placenta diversity. For example, it is not known whether ablation or mutation of ERVs is sufficient to cause substantial alteration in TF binding patterns during placentation. As noted above, due to the recent derivation of hTSCs and other human *in vitro* models, research on *cis*-regulatory elements in human trophoblast models is still rudimentary, awaiting intensive studies to unravel the core *cis*-regulatory elements required for human placenta development. Successful mapping of global hTSC-specific enhancers, as well as dynamic changes in enhancer usage during differentiation of hTSC toward ST and EVT, will help to explain the significant roles of enhancers in the maintenance and differentiation of hTSCs, and to identify TFs associated with the defined enhancers in hTSC and during ST or EVT differentiation *via* motif search, illuminating the mechanisms by which transcriptional regulatory landscape leads to trophoblast differentiation.

### Trans-Acting Factors Implicated in mTSCs and Placenta Development

In addition to *cis*-regulatory elements, identifying trophoblast/placenta-specific TFs and their global target genes is crucial to understanding how they form regulatory networks to modulate placenta-specific gene expression programs and to further elucidate the etiology of placenta malfunction-related complications. Conventional loss-of-function studies in mouse models identified a handful of key TFs in trophoblast lineages, including *Cdx2* (Strumpf et al., 2005), *Tead4* (Yagi et al., 2007), *Gata3* (Ralston et al., 2010), *Elf5* (Donnison et al., 2005), *Tfap2c* (Auman et al., 2002), *Eomes* (Russ et al., 2000), *Esrrb* (Luo et al., 1997), *Ets2* (Yamamoto et al., 1998), *Ascl2* (Guillemot et al., 1994), *Gcm1* (Anson-Cartwright et al., 2000), and *Hand1*(Riley et al., 1998). Notably, the vast majority of these genes are embryonic lethal upon their deletion, and phenotypes typically include severe defects in placenta development. While some TFs (*Elf5*, *Esrrb*, and *Tfap2c*) are known to be essential for the maintenance of mTSCs and during mouse placenta development, others (such as *Hand1* for trophoblast giant cells (TGC) and *Ascl2* for spongiotrophoblast) (Guillemot et al., 1994; Riley et al., 1998) are known to play central roles in mTSC differentiation toward more specialized trophoblast cell types, suggesting that different classes of TFs may play unique roles during trophoblast specification. Aligning with this, a recent mTSC-specific super-enhancer mapping approach has identified numerous novel TSC-specific TF candidates and classified them into four groups based on their expression patterns during mTSC differentiation, revealing that different classes of TSC-specific TFs play distinct roles in the maintenance or differentiation of mTSCs (Lee et al., 2019).

A recent forward genetic screen identified *Zfp281* as a mTSC-specific regulator, and an integrative analysis of global ZFP281 targets with global histone modifications disclosed that ZFP281 interacts with MLL or COMPASS complex mediating H3K4me3, suggesting that ZFP281 recruits the complex onto the mTSC-specific targets (Ishiuchi et al., 2019). Notably, ZFP281 is also a member of the mouse embryonic stem cell (ESC) core pluripotency network (Wang et al., 2006). As multiple mTSC-specific TFs, such as ESRRB, SOX2, and TFAP2C, are also members of the pluripotency network, it is reasonable to speculate that these TFs may have context-specific functions by forming distinct regulatory networks in mTSCs or mouse ESCs (mESCs) with context-specific interacting partner proteins, controlling different downstream target genes. In agreement with their roles in mTSCs, numerous studies reported that overexpression of TSC-specific TFs, such as *Arid3a* (Rhee et al., 2014), *Cdx2* (Niwa et al., 2005), *Gata3* (Ralston et al., 2010), *Tead4* (Nishioka et al., 2009), *Zfp281* (Ishiuchi et al., 2019), and *Fosl1* (Lee et al., 2018), could induce trophoblast-specific gene expression programs in mESCs. Mapping of the global binding sites of CDX2, ARID3A, and GATA3 in concert with RNA-seq and ATAC-seq during cell fate conversion revealed that these TFs initially repress ESC-specific genes and subsequently activate TSC-specific genes (Rhee et al., 2017). Similarly, global mapping of TFAP2A, TFAP2C, GATA2, and GATA3 combined with transcriptome analysis upon cell fate conversion of human ESCs (hESCs) to hTSLCs elucidated the binding landscape of these TFs during fate conversion. Among the TFs, GATA3 preferentially co-occupies its targets with other TFs and promotes the activation of placental genes, suggesting that GATA3 is a pivotal factor, and GATA2/3 and TFAP2A/C networks modulate early specification of trophoblast progenitors (Krendl et al., 2017). These observations ironically suggest that ESCs can also serve as a useful tool to study trophoblast development in various ways. Unfortunately, while the roles of more than hundreds of TFs have been elucidated in the field of mouse and human ESCs (and PSCs), only a few TFs have been functionally characterized in human trophoblast lineage specifications (Lee et al., 2007; Soncin et al., 2018; Saha et al., 2020). For a better understanding of early human trophoblast lineage differentiation, identification and validation of master TFs will be the first essential step. Furthermore, studies on how these key TFs collaboratively control the self-renewal of hTSCs, modulate differentiation toward ST or EVT, interact with their chromosomal target genes, and form regulatory networks with other interacting partner proteins will be tremendously important for deeper mechanistic understating of both normal placentation and diseased placenta.

### Epigenetic Regulations in mTSCs and Placenta

#### Histone Modifications

In addition to the enhancer-associated histone modifications (H3K27ac and H3K4me1) described above, genome-wide studies of active (H3K4me3 and H3K9ac) and repressive (H3K27me3 and H3K9me3) histone modifications suggested that epigenetic regulations also play important roles in development and early embryogenesis (Fogarty et al., 2015; Dahl et al., 2016; Liu et al., 2016; Xia et al., 2019). H3K4me3 is a well-known histone mark, generally observed at active promoters. H3K4 demethylase, KDM5B, is responsible for erasing these marks and plays a crucial role in mTSC differentiation toward specialized cell types by resetting the H3K4 methylation landscape at the promoters of mTSC self-renewal-related genes (Xu and Kidder, 2018). Bivalent domains that harbor both H3K4me3 and H3K27me3 marks are enriched near the promoters of inactive developmental genes in ESCs, and these marks allow for rapid activation of such genes upon developmental cues (Bernstein et al., 2006; Mikkelsen et al., 2007). Interestingly, it is still controversial whether TSCs harbor bivalent modifications, as one study reported that mTSCs have rare H3K27me3 domains (Rugg-Gunn et al., 2010), while another study showed 5,172 bivalent genes in mTSCs (Liu et al., 2016). Surprisingly, H3K27me3 and H3K4me2 ChIP followed by quantitative PCR in mTSCs revealed that bivalent marks are observed in the developmental genes (*Atoh1*, *Sox1*, *Hoxa7*, *Gata4*, and *Sox7*) that are not expressed in placenta development, while placenta-specific genes, such as *Cdx2, Pax3*, and *Hand1*, were only marked by H3K4me2. It has not been reported in hTSCs whether ST- or EVT-specific genes have bivalent domains and subsequently lose their bivalent signature upon differentiation, as well as how histone writers and erasers are regulated during ST or EVT differentiation. Studies on the dynamics of bivalent domains in the maintenance of hTSCs and their differentiation to ST or EVT in conjunction with global gene expression profiles will answer this question, and such approaches will further reveal how and to what extent the bivalent loci regulate genes required for trophoblast lineage commitment.

#### DNA Methylation

In general, DNA methylation at cytosine residues (5mC) plays crucial roles in cell-type-specific gene expression and silences transposons and other repetitive sequences on the genome (Li and Zhang, 2014). The genome of the trophoblast lineage is globally hypomethylated relative to that of somatic cells. In human CT, DNMT1, which is responsible for the maintenance of 5mC, is downregulated by promoter methylation, while upregulated DNMT3L plays important roles in placenta development by facilitating the activation of *de novo* methyltransferase DNMT3A and DNMT3B (Suetake et al., 2004; Arima et al., 2006; Novakovic et al., 2010). Abnormal DNA methylation during placenta development is known to be associated with pregnancy-related diseases (Koukoura et al., 2012), and methylation is also a pivotal regulatory mechanism underlying the mono-allelic expression of imprinting genes. DNMT3L is required for establishing maternal gene imprinting (Hata et al., 2002). Imprinted genes play essential roles in feto-placental development by affecting placental growth, morphology, and nutrient uptake capacity, as reviewed in-depth of imprinting mechanisms in murine placenta (Hanna, 2020). One fundamental question regarding the roles of DNA methylation is how DNA methylation contributes to trophoblast lineage commitment. Global methylation was investigated in human trophoblasts, including side-population trophoblast (trophoblast stem cell population), CT (intermediate progenitors), and EVT from the first trimester of the human placenta using reduced representation bisulfite sequencing (RRBS) (Gamage et al., 2018). Comparison of methylomes and transcriptomes revealed 41 hypomethylated genes are upregulated in EVT compared to CT and implicated in epithelial to mesenchymal transition (EMT) and metastatic cancer pathways. The results suggest that these 41 genes are responsible for the acquisition of an invasive phenotype of EVT, which is consistent with the fact that villous CT differentiates into invasive EVT through the EMT process observed in numerous invasive cancers (DaSilva-Arnold et al., 2015), further implying shared mechanisms underlying heightened invasive capabilities between trophoblasts and cancer cells. Therefore, it will be of great interest to investigate how dynamic alterations of global methylomes drive trophoblast lineage commitment. *In vitro* human trophoblast models including hTSCs would be an ideal system to capture the dynamics of global methylomes during hTSC differentiation to ST or EVT.

In addition to 5mC, 5hmC regulated by the 10–11 translocation factor, TET1, is essential for maintaining the self-renewing status of mTSCs (Chrysanthou et al., 2018). Therefore, testing the roles of TET1 in hTSC or their differentiation might be interesting. An integrative analysis of DNA 5mC, 5hmC, transcriptomes, and TET1 occupancy in mTSCs and differentiated mTSCs revealed that the ratio of 5hmC–5mC correlates with their target gene activity. Interestingly, most TET1 sites demarcate potential trophoblast enhancers while overlapping with active histone marks and TFAP2C binding sites (Senner et al., 2020). Multiple genome-wide methylation studies have been performed in human diseased placentas from patients experiencing recurrent pregnancy loss (RPL), identifying numerous differentially methylated regions (DMRs) associated with dysregulated genes (Du et al., 2020). Therefore, DNA methylation may contribute significantly to placenta pathology. The differences in methylation between male and female origin placentas have also been linked with susceptibility to pregnancy complications (Gong et al., 2018). Recently, DNA N^6^-methyladenine (6mA) modification was also reported in mammals (Xiao et al., 2018) and it was shown that 6mA contributes to epigenetic regulation by antagonizing the function of SATB1 during mTSC differentiation (Li et al., 2020). Conversely, recent comprehensive bioinformatics analyses of published data suggested that 6mA may not exist in mammals and this prior observation was due to false detection of 6mA (Douvlataniotis et al., 2020); therefore, more careful investigation is needed for the validation of 6mA contribution to mTSC differentiation and human trophoblast differentiation in the future.

### Chromatin Landscape in mTSCs and Placenta

The ATAC-seq approach in mTSCs has identified 57,019 accessible chromatin regions (Nelson et al., 2017). Moreover, a subsequent comparison of mTSC-specific open loci with those in different stages of the developing embryo revealed that approximately 20% of mTSC-specific open loci are also accessible in the 8-cell stage embryo and enriched with placenta-related genes, suggesting that a significant portion of putative mTSC enhancers is already open in the 8-cell stage during embryogenesis (Nelson et al., 2017). Dynamic changes in chromatin landscape were observed upon mTSC differentiation; upon differentiation, chromatin accessibility drastically increased at the genes associated with trophoblast lineage specification, although surprisingly, chromatin accessibility was not significantly changed for downregulated genes, suggesting the importance of chromatin openness changes for gene activation but not gene repression. Instead, the downregulated genes lost the occupancy of ESRRB, a TF for mTSC self-renewal, indicating that changes in chromatin accessibility are necessary but not sufficient for trophoblast gene regulation during differentiation (Nelson et al., 2017).

Recently, ATAC-seq for hTSCs derived from naïve hESCs has identified 12,132 open chromatin regions (Dong et al., 2020). A comparison of differentially accessible regions (DARs) between hTSCs and naïve hESCs revealed that the vast majority of DARs in hTSCs are located in loci that are distal from promoters, suggesting that long-range interactions between promoters and distal regulatory regions may be involved in transforming naïve hPSCs into hTSCs. However, comprehensive investigation of such long-range interactions has not yet been performed in hTSCs. Additionally, chromatin accessibility of EVT and ST has not been compared with that of hTSCs, and it remains unknown to what extent changes in accessibility facilitate cell fate specification in trophoblast lineages. Investigation of chromatin openness during differentiation of *in vitro* human trophoblast models will advance our understanding of how chromatin accessibility is altered in a different cellular context and contribute to the conversion of cellular identity during human trophoblast differentiation.

In addition to chromatin accessibility, chromatin architecture emerges as one of the key gene regulatory mechanisms to orchestrate spatiotemporal gene expression. Key cell-type-specific TFs are regulated by long-range looping between the promoter and enhancers. For instance, TEAD4 is robustly expressed in mTSCs (Nishioka et al., 2008, 2009). Circular chromosome conformation capture coupled with high-throughput sequencing (4C-seq) unveiled 64 putative long-range *Tead4* promoter interactomes in mTSCs. Comparison with enhancer histone signatures and open chromatin status revealed that five enhancer loci interact with the promoter of *Tead4* and significantly increase the activity of *Tead4* promoter in mTSCs, indicating that TSC-specific *Tead4* expression is regulated by inter-chromosomal promoter-enhancer interactions (Tomikawa et al., 2020). Of note, Hi-C in both mESCs and mTSCs revealed that enhancer-promoter interactions change dynamically between cell types and that the key mTSC-specific TFs tend to have promoter-enhancer interactions, particularly Tet1-regulated enhancers in mTSCs. These TFs are expressed relatively higher in mTSCs than in mESCs when they do not have promoter-enhancer interactions in mESCs, while mTSC-specific TFs that are suppressed in mESCs exhibit interactions between promoters marked with H3K27me3 in mESCs (Schoenfelder et al., 2018). Recent integrative analysis of H3K27me3 ChIP-seq with Hi-C and chromatin interaction analysis by paired-end sequencing (ChIA-PET) data discovered that long non-coding RNAs (lncRNAs), *Airn* and *Kcnq1ot1*, induce the spread of megabase-sized H3K27me3 domains in mTSCs by recruiting Polycomb repressive complex (PRC) to CpG islands (CGIs) in a lncRNA-dependent manner. This spreading of H3K27me3 is also reliant on the preexisting chromatin structure, abundance, and stability of *Airn* (Schertzer et al., 2019). Since these chromatin interaction studies in mTSCs revealed the dynamics and significance of chromatin structure in determining cell-type specificity, similar research using multiple human trophoblast models under the conditions of self-renewing and ST or EVT differentiation will also help to define how and to what extent chromatin architecture contributes to human placenta development.

### The Differences Between Human and Mouse Placenta Development

Mouse and human placentas have many commonalities, as both of them are discoid and hemochorial placentas, and they also have many functionally conserved genes. However, there are also many apparent differences between them, such as size, shape, cellular organization, gestational length, and overall structure (Rossant, 2015; Schmidt et al., 2015). Accordingly, mouse placenta models do not always perfectly mimic human pregnancy disorders such as PE (Bodnar et al., 2005; Aubuchon et al., 2011). It is often difficult to extrapolate findings from studies on rodent placentation. A growing body of evidence indicates that human placenta development differs from that of mouse spatiotemporally. For example, human blastocysts show OCT4 expression restricted to the inner cell mass (ICM) about 2 days later than the mouse. While mouse blastocysts show mutually exclusive expression patterns between *Oct4* (in the ICM) and *Cdx2* (in the TE), *CDX2* in the human blastocyst is initially co-expressed with *OCT4*, the latter of which is not confined to the ICM until just before implantation (Niakan and Eggan, 2013). Core TE-specific TFs (*Id2*, *Elf5*, and *Eomes*) are exclusively expressed in mouse trophoblast lineages, whereas human orthologs are not present in human TE (Blakeley et al., 2015), but *ELF5* expression is observed in the trophoblast subpopulations of early implanted embryos (Hemberger et al., 2010; Soncin et al., 2018). In particular, genome-wide transcriptome comparison between human and mouse placentas not only unveiled that the itinerary of mouse placental development is parallel to only the first half of gestation in the human placenta, but also revealed significant differences in transcriptomes and identified VGLL1 as a human-specific marker for CT (Soncin et al., 2018).

Furthermore, immunohistochemistry in human placental tissues showed that a short isoform of *TP63* is specific to the human rather than the mouse placenta. *TP63* is mainly expressed in primary CT, and its expression level decreases when the cells undergo differentiation into either ST or EVT (Lee et al., 2007). Despite considerable differences between human and mouse placentation, only a few human placenta-specific TFs and *cis*-regulatory elements have been identified, and little is known about their mechanisms in regulating human trophoblast fate. It is still ambiguous to what extent key TFs share common targets and play conserved roles between humans and mice. Considering the differences between mouse and human placenta (Soncin et al., 2015), it is essential to identify human-specific *cis*-regulatory elements and TFs. All the above evidence highlights the necessity of the use of *in vitro* human placenta models. The multiple omics approaches in human *in vitro* trophoblast modes will reveal human-specific regulatory elements and factors as well as distinct epigenetic regulations that can then later be validated for possible roles in human placenta development or pathology.

## *In vitro* Models of Human Placenta Development

Although rodent models, especially mouse *in vivo* and *in vitro* model systems, have been widely used in the field, animal model systems do not completely mimic the human placenta in various ways, as described above. Before establishing *bona fide* hTSCs, choriocarcinoma, immortalized cells, and primary CT in the placenta were used as *in vitro* models of human placenta development (Nagamatsu et al., 2004; Bilban et al., 2010; Dietrich et al., 2020). However, there were multiple drawbacks. Carcinoma cells do not completely recapitulate the multipotent human trophoblast, and they show an abnormal gene expression profile. Conversely, although multipotent, access to primary CT is limited, and they do not proliferate *in vitro* (Kliman et al., 1986; Soares et al., 2018). Due to these issues, more recent efforts have been made to establish human trophoblast models from human PSCs [hESCs or human induced pluripotent stem cells (hiPSCs)]. Since then, rapid advances in the field have established multiple human *in vitro* placenta models from various sources and using different protocols (Figure 1). In this section, we discuss recently available *in vitro* model systems of early human placenta development and also describe their applications and limitations.

### BMP4-Induced hTSLCs

Since it has been reported that bone morphogenetic protein 4 (BMP4) can initiate differentiation of hESCs toward trophoblast lineages (Xu et al., 2002), multiple groups have attempted to convert hESCs to hTSLCs by treating them with BMP4 alone or in conjunction with small molecules, such as BAP (BMP4, A83-01, and PD173074) (Amita et al., 2013; Li et al., 2013; Horii et al., 2016). BMP4 treatment in the absence of FGF2 induces morphological changes of hESCs to epithelial cells that express KRT7 within 48 h (Amita et al., 2013). These cells robustly expressed trophoblast-specific markers, including HLA-G and secreted placenta hormones, such as chorionic gonadotropin, progesterone, and placental lactogen. Another study showed that CDX2+/TP63+CT-like cells, which have the potential to differentiate into ST- and EVT-like cells (bipotency), can be derived from hPSCs by the treatment of BMP4-containing defined media, based on the marker gene expression, hormone secretion, and invasion capacity (Horii et al., 2016). Although the exact mechanistic roles of BMP4 in the transformation of hPSCs to hTSLCs have not been well characterized, depletion of TP63 impaired the conversion of hPSCs to functional trophoblasts (Li et al., 2013), implying that BMP4 functions through TP63. These studies suggest that BMP4 may initiate the activation of trophoblast-specific gene expression in hPSCs that are known to harbor relatively loose chromatin structures (Melcer and Meshorer, 2010; Amita et al., 2013). Notably, BMP4-induced hTSLCs cannot proliferate for a prolonged time, and they ultimately differentiate into multiple uncharacterized cell populations, suggesting BMP4 treatment alone is not sufficient to convert hPSCs to self-renewing hTSCs with canonical bipotency. Nevertheless, these BMP4-induced hTSLCs have multiple advantages over other models. Since hiPSCs can be used as starting cells in addition to preexisting hESCs, generation of patient-specific hTSLCs is feasible by sequential establishment of hiPSCs from patients’ somatic cells followed by conversion of the hiPSCs to hTSLCs. In turn, the approaches using trisomy 21 hPSCs and PE-derived hiPSCs successfully model trophoblast differentiation defects (Horii et al., 2016, 2021). Since many hiPSC lines have been established or can be established from human patients with various symptoms, similar approaches can be employed to understand previously unknown links between human diseases and the events during early placentation.

### Bona Fide hTSCs

In 2018, Okae et al. established multiple self-renewing hTSCs lines harboring bipotency from the first-trimester placenta as well as human blastocysts (Okae et al., 2018). This long-standing goal of the field was achieved by the manipulation of multiple signaling pathways (activation of the epidermal growth factor (EGF) and Wnt signaling pathways, along with inhibition of the transforming growth factor beta (TGFB) pathway) combined with HDAC inhibitors and Rho-associated protein kinase (ROCK) inhibitor treatment. Although hTSCs present an excellent *in vitro* placenta model system and reliable protocols to differentiate them into ST and EVT are available, access to blastocysts or primary CT from the first-trimester placenta is still limited due to ethical issues. Interestingly, multiple research groups have recently reported the successful conversion of naïve hPSCs to hTSLCs (Cinkornpumin et al., 2020; Dong et al., 2020) using the defined culture condition used for the derivation of bona fide hTSCs (Okae et al., 2018). Notably, BMP4 treatment on naïve PSCs leads to adverse effects, such as promoting cell death, implying that naïve hPSCs must exit the naïve state to the primed state to efficiently respond to BMP4 for the conversion from hESCs to hTSLCs. Conversely, cultivating naïve hPSCs on Collagen IV in hTSC media allowed for the establishment of hTSLCs, whereas primed hPSCs cannot be transformed toward hTSLCs under the same conditions (Dong et al., 2020). Comparable to genuine hTSCs, naïve hPSC-derived hTSLCs can be maintained for over 20 passages without losing their bipotency, and they show overall gene expression signatures similar to hTSCs as well. Another group also reported that human expanded potential stem cells (hEPSCs) can be converted into hTSLCs under the bona fide hTSC culture condition (Gao et al., 2019). The hTSLCs derived from hEPSCs also showed bipotency; however, they did not show intact self-renewal capacity.

In contrast to the previous failures to generate self-renewing hTSLCs from primed hPSCs using BMP4 (Amita et al., 2013; Li et al., 2013; Horii et al., 2016), two independent groups have demonstrated the successful conversion of primed hESCs to hTSLCs. One group showed that the culture of hiPSCs on a nickel micromesh with triangular shapes results in cystic cells having characteristics of hTSCs in the absence of BMP4 (Li et al., 2019). These cells proliferated for over 205 days and showed bipotency, suggesting that it may be feasible to convert primed hPSCs to hTSLCs without BMP4. More recently, another study showed that the culture of hESCs in chemically defined media containing BMP4, SB43154, and S1P (the phospholipid sphingosine 1-phosphate) could convert primed hPSCs to two distinct subpopulations of hTSLCs (CDX2+ and CDX2- hTSLCs) (Mischler et al., 2021). Although additional functional validations are required to understand the true nature of all these hTSLCs, the results at least showed that different culture conditions can be used to transform naïve or primed hPSCs to hTSLCs, suggesting that there may be multiple molecular paths for the conversion of hPSCs toward hTSLCs.

### iTSCs, TF-Mediated Conversion Models

In addition to the establishment of hTSLCs by applying defined culture conditions, recent studies showed that reprogramming of human somatic cells to human iTSCs by ectopic expression of TFs is feasible (Castel et al., 2020; Liu et al., 2020). Notably, unlike mouse iTSCs derived from the overexpression of multiple trophoblast-specific TFs (EOMES, GATA3, TFAP2C, and MYC or ETS2) in mouse fibroblasts (Benchetrit et al., 2015; Kubaczka et al., 2015), human iTSCs were established by reprogramming somatic cells with the induction of OCT4, SOX2, KLF4, and MYC (original TFs used to generate iPSCs; Takahashi and Yamanaka, 2006; Takahashi et al., 2007; Park et al., 2008) coupled with hTSC culture conditions. In this approach, expansion of the cells obtained from the intermediate stage of reprogramming in hTSC media was sufficient to reprogram somatic cells toward human iTSCs with the full differentiation potential of early trophoblasts without ectopic expression of trophoblast-specific TFs. This surprising result suggests that at least a small portion of cells during the intermediate stage of reprogramming harbor active trophoblast-specific gene expression programs, and the hTSC culture condition may stabilize hTSC-specific gene expression programs by repressing upregulation of other lineage-specific genes. It would be interesting to investigate the mechanisms underlying this observation and whether mouse iTSCs can be derived with the same procedure through which human iTSCs were derived from somatic cells.

### 3D Culture Models

Cells cultured on 2-dimensional (2D) surfaces do not always accurately recapitulate authentic tissue environments where cells are spatially surrounded by other cells in 3-dimensions (3D). Therefore, it is of great interest to study 3D-cultured cells that behave more closely to *in vivo* tissue (Simian and Bissell, 2017). Recently, multiple independent groups established placenta organoid models. One group generated trophoblast organoids *via* 3D culture of first-trimester villous CT (Haider et al., 2018). These trophoblast organoids showed globally similar gene expression profiles to the primary CT. Under self-renewing conditions, the organoids are composed of CT (outside) and ST (inside) that are spontaneously differentiated from CT, whereas CT can further differentiate into HLA-G + EVT at the outer CT layers upon withdrawal of Wnt stimulators. Another group also established genetically stable trophoblast organoids that can differentiate into both ST and EVT from EPCAM + proliferative trophoblasts (Turco et al., 2018). These organoids form villous-like structures where the basement membrane is located outside the organoids, whereas syncytial masses reside in the central cavity. These organoids can secrete placenta-specific hormones, growth factors, and glycoprotein, and can further differentiate into EVT. These trophoblast organoids are potentially useful models for studying critical elements required for placenta morphogenesis, feto-maternal communication, and transmission of pathogens. Most recently, hTSC-derived trophoblast organoids were also reported (Saha et al., 2020). Like CT-derived organoids, hTSC organoids had villous-like structures. Interestingly, depletion of TEAD4 in hTSCs prevented organoid formation. Accordingly, hTSCs derived from RPL placentas showing a reduced level of TEAD4 failed to efficiently form trophoblast organoids, suggesting a significant role of TEAD4 in human placenta development and RPL placentas.

## Conclusion and Perspectives

Recent genome-wide identification of *cis*-regulatory elements and *trans*-acting factors in mTSCs and placenta models revealed multiple essential factors and their action mechanisms underlying mTSC maintenance and trophoblast lineage specification. Also, in combination with conventional genetics approaches, global inspections of histone signatures, DNA methylation, chromatin openness, and 3D architecture in mTSC models unveiled that placenta development is orchestrated by multiple genetic and epigenetic modulators whose defects might be associated with pregnancy complications. Although both mouse *in vivo* and *in vitro* models have advanced our understanding of the mechanisms underlying placenta development, fundamental discrepancies between human and mouse placentas have been a major issue. Recent success in establishing multiple human *in vitro* placenta models, such as hTSCs, hTSLCs, iTSCs, and 3D trophoblast organoids, may fill the gap of knowledge by providing tremendous opportunities to study human trophoblast and placenta-specific gene regulation. In addition, they will serve as useful *in vitro* models for both normal and abnormal human placentation, especially in the early stages of development.

Despite recent advances in developing research and diagnostic tools, the placenta remains one of the least understood organs in the human body. Since multiple human *in vitro* model-based approaches have recently become available, numerous questions can be addressed: What factors or regulators are responsible for the specification of various cell types in the human placenta? How do they form regulatory networks modulating human trophoblast cell-type-specific gene expression programs? How do the malfunctions of the key regulators trigger placenta-associated complications? How do trophoblasts collaborate with maternal immune cells for a successful pregnancy without immunological aggression? Is it possible to successfully derive multiple functional cell types from hTSCs, hTSLCs, iTSCs, or 3D organoid models? Recently developed multiple omics approaches with human *in vitro* placenta models, possibly from both normal and diseased placentas, will provide us with a more holistic view of genetic and epigenetic regulatory mechanisms in placentation and etiologies of placenta-associated pregnancy complications.

## Author Contributions

B-KL and JK wrote the manuscript. Both authors contributed to the article and approved the submitted version.

## Conflict of Interest

The authors declare that the research was conducted in the absence of any commercial or financial relationships that could be construed as a potential conflict of interest.

## Abbreviations

ATAC-seq: transposase-accessible chromatin followed by sequencing
BAP: BMP4, A83-01, and PD173074
BMP4: bone morphogenic protein 4
ChIP-seq: chromatin immunoprecipitation coupled with high-throughput sequencing
CT: cytotrophoblasts
EGF: epidermal growth factor
EMT: epithelial to mesenchymal transition
ESCs: embryonic stem cells
EVT: extravillous trophoblasts
HDAC: histone deacetylase
hEPSCs: human expanded potential stem cells
Hi-C: A method to study the three-dimensional architecture of genomes
IUGR: intrauterine growth restriction
iPSCs: induced pluripotent stem cells
iTSCs: induced trophoblast stem cells
KO: knockout
5mC: DNA methylation at cytosine residues
6mA: DNA methylation at adenine residues
NGS: next-generation sequencing
PE: preeclampsia
PSCs: pluripotent stem cells
ROCK: rho-associated protein kinase
RPL: recurrent pregnancy loss
RRBS: reduced representation bisulfite sequencing
S1P: phospholipid sphingosine 1-phosphate
ST: syncytiotrophoblasts
TE: trophectoderm
TFs: transcription factors
TGC: trophoblast giant cells
TGFB: transforming growth factor beta
TSCs: trophoblast stem cells
TSLCs: trophoblast stem (TS)-like cells.
